# Evaluating and Improving Syndrome Differentiation Thinking Ability in Large Language Models: Method Development Study

**DOI:** 10.2196/75103

**Published:** 2025-06-20

**Authors:** Chunliang Chen, Xinyu Wang, Ming Guan, Wenjing Yue, Yuanbin Wu, Ya Zhou, Xiaoling Wang

**Affiliations:** 1 East China Normal University, No.3663 Zhongshanbei Road, Shanghai, China, 86 18621306726; 2 Shanghai Institute of Intelligent Science and Technology, Tongji University, Shanghai, China; 3 Guangxi Key Laboratory of Trusted Software, Guilin University of Electronic Technology, Guilin, China

**Keywords:** large language model, traditional Chinese medicine, RAG, instruction tuning, TCM LLMs, syndrome differentiation thinking

## Abstract

**Background:**

A large language model (LLM) provides new opportunities to advance the intelligent development of traditional Chinese medicine (TCM). Syndrome differentiation thinking is an essential part of TCM and equipping LLMs with this capability represents a crucial step toward more effective clinical applications of TCM. However, given the complexity of TCM syndrome differentiation thinking, acquiring this ability is a considerable challenge for the model.

**Objective:**

This study aims to evaluate the ability of LLMs for syndrome differentiation thinking and design a method to effectively enhance their performance in this area.

**Methods:**

We decomposed the process of syndrome differentiation thinking in TCM into three core tasks: pathogenesis inference, syndrome inference, and diagnostic suggestion. To evaluate the performance of LLMs in these tasks, we constructed a high-quality evaluation dataset, forming a reliable foundation for quantitative assessment of their capabilities. Furthermore, we developed a methodology for generating instruction data based on the idea of an “open-book exam,” customized three data templates, and dynamically retrieved task-relevant professional knowledge that was inserted into predefined positions within the templates. This approach effectively generates high-quality instruction data that aligns with the unique characteristics of TCM syndrome differentiation thinking. Leveraging this instruction data, we fine-tuned the base model, enhancing the syndrome differentiation thinking ability of the LLMs.

**Results:**

We collected 200 medical cases for the evaluation dataset and standardized them into three types of task questions. We tested general and TCM-specific LLMs, comparing their performance with our proposed solution. The findings demonstrated that our method significantly enhanced LLMs’ syndrome differentiation thinking. Our model achieved 85.7% in Task 1 and 81.2% accuracy in Task 2, surpassing the best-performing TCM and general LLMs by 26.3% and 15.8%, respectively. In Task 3, our model achieved a similarity score of 84.3, indicating that the model was remarkably similar to advice given by experts.

**Conclusions:**

Existing general LLMs and TCM-specific LLMs continue to have significant limitations in the core task of syndrome differentiation thinking. Our research shows that fine-tuning LLMs by designing professional instruction templates and generating high-quality instruction data can significantly improve their performance on core tasks. The optimized LLMs show a high degree of similarity in reasoning results, consistent with the opinions of domain experts, indicating that they can simulate syndrome differentiation thinking to a certain extent. These findings have important theoretical and practical significance for in-depth interpretation of the complexity of the clinical diagnosis and treatment process of TCM.

## Introduction

In recent years, the large language model (LLM) has made significant progress in electronic medical record analysis [1], drug discovery [2], clinical decision support [3], and other medical fields [4,5]. As an important part of the health care system, traditional Chinese medicine (TCM) is known for its unique theoretical framework and complex knowledge system [6]. The LLMs offer better means to explore and understand knowledge from classic texts, prescription data, and clinical cases, laying the foundation for the clinical application of TCM.

The core concept of TCM diagnosis and treatment emphasizes a dynamic treatment strategy based on patient characteristics, which requires the LLMs to have profound medical knowledge and adaptive reasoning ability. To address the specificity of TCM-domain knowledge, the researchers have tried to improve the model performance through domain fine-tuning by leveraging publicly available datasets, including classical TCM literature, educational materials, and clinical case records [6-8].

Some models have also integrated domain-specific pathology data such as TCM formulas and medical record files to enhance specialization and specificity. For example, BianCang [9] added clinical data on cardiology and used a two-stage training method of injecting domain knowledge and alignment to obtain results. Yang et al [10] developed ZhongJing by leveraging a Chinese multi-turn medical conversation dataset of 70,000 authentic doctor-patient dialogues, enabling the model to better capture the nuances and complexity of clinical communication.

Although existing TCM-specific LLMs demonstrated improved performance in some areas of TCM, their effectiveness in handling complex TCM tasks remains inadequate. This limitation stems from the lack of targeted reasoning training for TCM syndrome differentiation thinking. The highly context-dependent and flexible nature of TCM diagnostic reasoning necessitates a deep understanding of the intricate logic underlying disease patterns, placing higher demands on the model’s comprehension and reasoning ability [11,12]. LLMs often struggle with knowledge confusion when confronted with the nuanced reasoning required for TCM syndrome differentiation. For instance, the principle of “different treatments for the same disease” is common in TCM, where varying treatment approaches and herbal prescriptions are recommended for the same condition across different texts. Such inconsistencies in the training data significantly hinder the reasoning ability of LLMs in TCM. It is worth noting that Chain-of-Thought (CoT)–based mechanisms exhibit clear limitations in this field, as the reasoning explanations generated by these models frequently devolve into nonsensical or irrelevant outputs [13]. In contrast, the direct application of Retrieval-Augmented Generation (RAG) can provide accurate contextualized hints derived from professional TCM knowledge [14].

To evaluate the capacity of LLMs for performing TCM syndrome differentiation, we developed three core tasks grounded in the foundational theories of TCM: pathogenesis inference (Task 1), syndrome inference (Task 2), and diagnostic suggestion (Task 3). For the evaluation dataset, we extracted 200 medical cases from *Essence of Modern Chinese Medicine Case Studies* [15] and standardized these cases into the corresponding task formats. Furthermore, we developed a methodology for generating instruction data based on the idea of an “open-book exam.” Given each task’s unique logic, we designed a specialized data template for each task type. To enhance the model’s comprehension of the problem, we extracted relevant information from a local knowledge base and inserted it into predefined positions within the templates. Thus, we generated 800 high-quality instruction samples for fine-tuning the model using this approach. This method of embedding RAG content into instruction data enables the model to better understand contextual information unlike models that directly input RAG content.

## Methods

### Overview

To enhance the ability of existing LLMs in TCM syndrome differentiation thinking, this study proposed an instruction data generation method based on the idea of an “open-book exam.” Open-book exams allow candidates to consult materials during the exam, but only those students who have mastered how to obtain and interpret the answers in advance quickly can cope with them efficiently. Similarly, we treated each type of TCM task as an “open-book exam.” Incorporating domain knowledge into the instruction template enhances the LLMs’ ability to reason information from the materials, enhancing their performance in TCM tasks. The method consists of three core parts: (1) designing core tasks based on TCM theory; (2) constructing domain-specific instruction data templates for different task categories; and (3) designing an evaluation method to compare and analyze LLMs.

### Task Decomposition

According to the TCM pathogenesis theory, the clinical process of TCM syndrome differentiation thinking requires deducing from symptoms to syndromes by analyzing the pathogenesis factors of the surface-level symptoms one-by-one and comparing the relationships between the factors. For clinical application, in addition to analyzing the pathogenesis factors, the patient’s judgment also depends on understanding the integrated relationships among these factors. Based on this framework, we have extracted three core tasks: pathogenesis inference, syndrome inference, and diagnostic suggestion. The decomposition of the complex process of TCM identification into clear task modules helps the model in hierarchical analysis of the TCM identification ideas. The three tasks are explained in Table 1.

**Table 1.** Comparison of the performance of different models in three tasks.

Task	Explanation	Question format
Task 1: Pathogenesis inference	Pathogenesis refers to the mechanism of disease occurrence, development, change and outcome, including the changes and mechanisms of etiology, nature, location, and course of disease.	Select the most appropriate pathogenesis based on the clinical information. You will choose the most correct answer from options A, B, C, D, E, F, G, H, I, and J. The questions are as follows: “Clinical Information” “Pathogenesis Options”
Task 2: Symptom inference	Symptoms are a pathological summary of the body’s overall response to a disease at a particular stage.	Select the most appropriate syndrome based on the clinical information. You will choose the most correct answer from options A, B, C, D, E, F, G, H, I, and J. The questions are as follows: “Clinical Information” “Syndrome Options”
Task 3: Diagnostic suggestion	Diagnostic suggestion forms a comprehensive understanding of the disease’s nature. This step emphasizes summarizing the local to the whole and from phenomenon to the essence, thus guiding the formulation of treatment plans.	Write a summary and syndrome differentiation analysis based on the clinical information, core pathogenesis and syndrome. The questions are as follows: “Clinical Information” “Core Pathogenesis” “Core Syndrome”

### TCM Instruction Data Generation

We treated each TCM task as an open-book exam scenario, where structured domain knowledge is embedded directly into the prompt templates, in a manner similar to the process of simulating a test-taker’s responses on an exam. The model was expected to learn to use this information in specific contexts to generate accurate responses.

**Figure 1.**
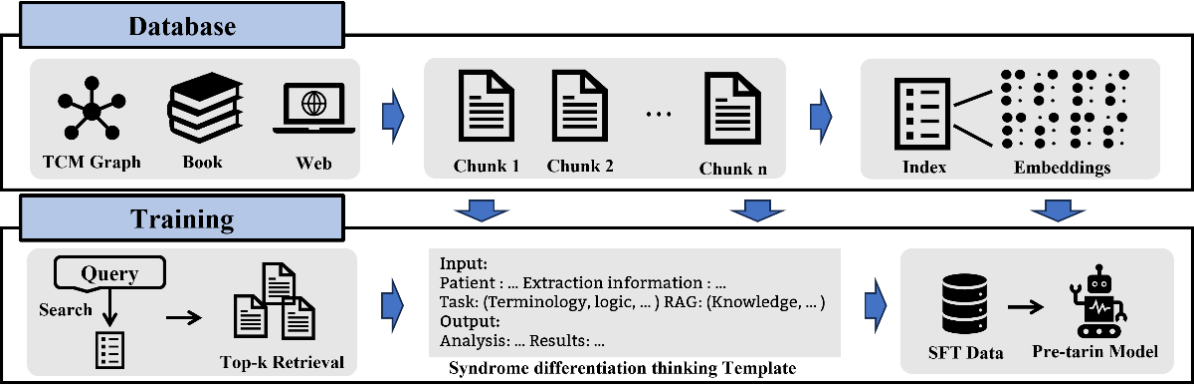
A framework for generating instruction data is formed by integrating the local knowledge base into the Syndrome Differentiation Thinking Template.

As illustrated in Figure 1, the local knowledge base was partitioned into distinct segments, referred to as chunks, and vector models were employed to generate embeddings. Let Q denote the question to be addressed. The knowledge base is represented as $D=\{d_{1},d_{2},\cdots ,d_{n}\}$, where $d_{i}$ corresponds to a chunk of the document. Embeddings for each chunk are generated using a vector model, denoted as $E=\{e_{1},e_{2},\cdots ,e_{n}\}$. Each chunk embedding is defined as $e_{i}=f(d_{i})$, with f representing the embedding generation function. The similarity matching is established through cosine similarity, defined as follows:


$$sim\left(e_{i},Q\right)=\frac{e_{i}\cdot Q}{\left\|e_{i}\right\|\left\|Q\right\|}$$

where $\left\|∙\right\|$represents the vector norm, and chunks exhibiting the highest similarity are selected to form the matched document set $D^{`}$:


$$D^{\prime }=\left\{d_{i}\in D\;|\;∼(e_{j},Q)\geq \theta \right\}$$

In this expression, θ represents a predefined similarity threshold. The reference knowledge is then integrated into the instructional data I§lt;Input,Output§gt;, thereby enhancing reasoning capabilities across three tasks during practical evaluations. To incorporate domain-specific knowledge into the reasoning process, we introduce a structured template T, parameterized as $Input=T\left[Q,D^{`}\right]$.

### Data Collection

We analyzed 200 medical cases from *Essence of Modern Chinese Medicine Case Studies* [15] and systematically organized the data into categories, including patient demographics, clinical manifestations, tongue and pulse diagnoses, etiologic factors, and diagnostic examination findings. To ensure data consistency and accuracy, nonessential or irrelevant details were excluded during the extraction process, retaining only critical information related to clinical reasoning. An illustrative example is presented in Textbox 1.

Textbox 1. **Example medical case extracted from books.**

The patient is a woman, 18-years-old. Sudden decline in vision of both eyes for 10 days, and blindness in the left eye for 3 days. The patient has a dull headache in the forehead, pulling pain behind the eyeball when moving the eye.

Examination: Visual acuity–Right eye 0.02, no light perception in the left eye. Moderate dilation of pupils in both eyes, sluggish light reflex in the right eye, and absence of direct light reflex in the left eye. Intraocular pressure is 18.86 mmHg in both eyes. The optic discs of both eyes are congested, mildly elevated, with blurred boundaries, disappearance of physiological cupping, dilated retinal veins, and mild edema of the retina near the optic disc in the left eye.

Western medical diagnosis: Acute optic neuritis (both eyes)

Examination as per Traditional Chinese medicine: Depressed mood, dizziness, blurred vision, flank pain, bitter taste in the mouth, and chest tightness; the tongue is red with thin yellow coating, and the pulse is wiry

Pattern differentiation: Sudden blindness (both eyes) – type of liver qi stagnation and obstruction of collaterals.

Treatment principle: It is appropriate to soothe the liver and resolve stagnation, and to unblock the channels and invigorate the collaterals. The prescription used is Shugan Mingmu decoction (an empirical formula).

Commentary: Sudden blindness refers to serious internal ocular disorders where the external appearance of the eye seems normal but there is a sudden and drastic decline in vision, or even blindness, in one or both eyes. Although the external appearance of the affected eye shows no obvious abnormalities, various pathological changes occur inside the pupil, and the etiology and pathogenesis are more complex. Due to the acute onset, early treatment should be initiated. In western medicine, various fundus diseases can cause sudden blindness, the most common being central retinal vascular occlusion and acute optic neuritis.

To enable automatic quantitative scoring, Tasks 1 and 2 were designed as multiple-choice questions, with all disease mechanisms and clinical evidence data aggregated into a standardized dataset. The answer options for these questions were generated by randomly permuting the original option set, with distractor options created by systematically rearranging plausible alternatives. While Task 3 was formatted as open-ended questions, answers were derived from source text, with scores calculated via cosine similarity between answer embeddings.

### Ethical Considerations

This study used data from published medical records in publicly available literature, which did not involve human subjects or contain personally identifiable information. Accordingly, ethics committee approval was not required.

### Experimental Setup

The base model used in this study was InternLM2-Chat2-20B-SFT [16], which has been pretrained on domain-specific knowledge from TCM. This enables the model to effectively address complex medical scenarios and perform basic TCM question-and-answer tasks without requiring further fine-tuning. Text embeddings were generated using Acge-Text-Embedding [17]. For general LLMs, we used the official API interface, while for TCM LLMs, we deployed reasoning locally. Specifically, the experiments were conducted on a single NVIDIA A6000 GPU (48 GB memory). All model weights were publicly available via the Hugging Face Hub. Instruction tuning was performed using the Llama Factory framework [18], with LoRA integration. The hyperparameters were configured with a $learning\;rate=2\times 10^{-4}$ and training epochs set to 5. After fine-tuning, parameter consolidation of the optimized modules was sufficient for deployment.

## Results

We establish scoring criteria for each task. For Tasks 1 and 2, scores were calculated using a proportional multiple-choice method, computed as the ratio of correct responses to the total number of options presented. For Task 3, the cosine similarity between the model output and the standard output was determined. The resulting similarity scores were then linearly scaled to a range of 0‐100, with higher values indicating greater similarity.

As demonstrated in Table 2, our model achieved superior performance across all three tasks, with accuracy rates of 85.7% and 81.2% for Task 1 and Task 2, respectively, and a similarity score of 84.3 for Task 3. In contrast, the powerful general model, ChatGPT4 performed poorly in Task 1 and Task 2, with accuracy rates of only 24.4% and 36.3%, respectively, but achieved a score of 78.2 in Task 3. Task 3 is an open-ended generative task, evaluated using cosine similarity, which emphasizes linguistic fluency and semantic coherence rather than factual accuracy. This evaluation criterion aligns well with ChatGPT-4’s strength in generating natural and coherent language, even when its underlying understanding of TCM is limited. In contrast, Tasks 1 and 2 demand a deeper comprehension of specific TCM concepts, where the lack of specialized training in TCM becomes more evident in ChatGPT-4’s performance. Additionally, DeepSeek-v3 and Qwen-max demonstrated more balanced performance across multiple tasks, with scores consistently exceeding the range of 50% to 60%, likely due to their targeted training in TCM-related tasks. Our model demonstrates strong advantages in both accuracy and similarity across structured TCM tasks, highlighting its effectiveness in domain-specific reasoning and output generation.

**Table 2.** Comparison of the performance scores of different models in three tasks.

Models	Task 1,^a^ (%)	Task 2,^b^ (%)	Task 3^c^ (score)
ChatGPT4 [19]	24.4	36.3	78.2
DeepSeek-v3 [20]	48.8	57.7	53.4
Qwen-max [21]	57.1	63.6	79.7
InternLM3-Latest [22]	53.1	62.5	62.4
ChatGLM4 [23]	36.5	39.5	79.2
Our study	85.7	81.2	84.3

a Task 1: Pathogenesis inference.

b Task 2: Symptom inference.

c Task 3: Diagnostic suggestion.

## Discussion

### Comparison With Other Works

To better contextualize our proposed TCM LLM, we compare it with four representative models: ShenNong [24], BianCang [9], HuangDi [25], and ZhongJing [10]. These models were selected due to their prominence in the TCM domain, publicly available methodologies, and diverse approaches to integrating TCM knowledge into LLMs. Each model reflects a distinct strategy in data sourcing, architectural design, or application focus, making them suitable benchmarks for evaluating our method. Specifically, ShenNong compiles an extensive dataset of TCM information and generates a knowledge graph, which serves as a benchmark for creating over 110,000 TCM-related instruction data points to train a foundational model. BianCang integrates a vast collection of ancient TCM texts, modern educational materials, and clinical data, offering users precise TCM knowledge quizzes, diagnostic support, and treatment recommendations. HuangDi is built on the Ziya-LLaMA-13B-V1 base model and incorporates TCM textbooks, website data, and other TCM corpora, demonstrating a notable comprehension of TCM knowledge. ZhongJing enhances the model’s proficiency in TCM clinical diagnosis and treatment by training it on classical TCM literature.

As demonstrated in Table 3, BianCang outperforms all other TCM LLMs by achieving accuracy rates of 59.3% and 65.4% on Tasks 1 and 2, respectively, and scoring 69.6 on Task 3. Although BianCang benefits from training on case diagnosis data, thereby enhancing its understanding of syndrome differentiation, it still underperforms compared to our proposed solution across all tasks. These results suggest that relying exclusively on domain-specific training data proves inadequate and significantly constrains a model’s capacity for TCM reasoning. These results underscore that our approach not only outperforms existing TCM LLMs but also represents a meaningful advancement in the integration of domain knowledge with deeper reasoning capabilities.

**Table 3.** Performance comparison of TCM LLMs across multiple tasks.

Models	Task 1,^a^ (%)	Task 2,^b^ (%)	Task 3,^c^ (score)
ShenNong	42.2	53.3	42.8
BianCang	59.3	65.4	69.6
HuangDi	32.4	45.6	52.4
ZhongJing	38.1	36.5	59.6
Our study	85.7	81.2	84.3

a Task 1: Pathogenesis inference.

b Task 2: Symptom inference.

c Task 3: Diagnostic suggestion.

To more systematically evaluate the performance advantages of the proposed method, we also selected two popular LLMs enhancement methods for comparison: CoT, and RAG. Specifically, CoT significantly improves the model’s logical reasoning ability in a step-by-step manner by guiding it to gradually generate intermediate reasoning steps, thereby showing higher interpretability and accuracy in complex tasks. On the other hand, RAG expands the model’s knowledge coverage and enhances its contextual understanding and domain adaptability by dynamically retrieving and integrating external knowledge during generation. We retained the base model without any enhancement methods as the baseline control group, referred to as Base, to comprehensively measure the relative performance improvements across different methods.

**Figure 2.**
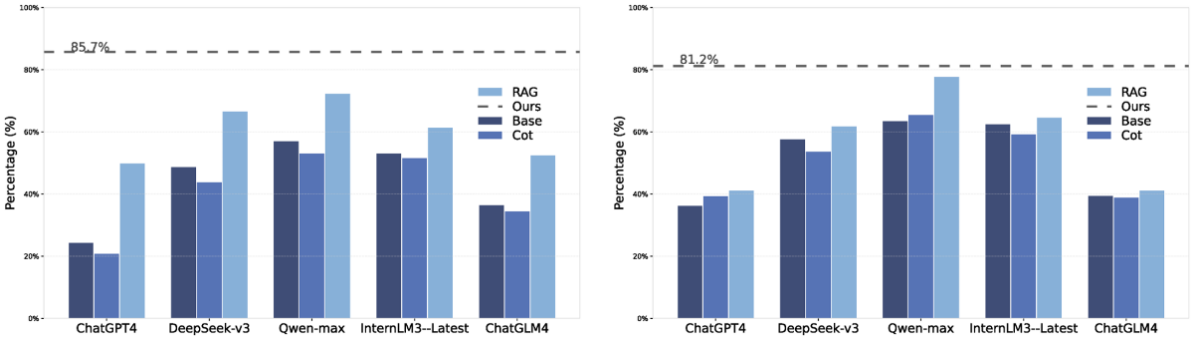
Performance comparison of RAG, CoT, and Base models across Two Tasks; Task 1 pathogenesis inference (left), Task 2 symptom inference (right). CoT: Chain-of-Thought; RAG: Retrieval Augmented Generation.

As illustrated in Figure 2, RAG demonstrated significant performance advantages in both Task 1 and Task 2. The accuracy of all models using RAG exceeded that of the Base and CoT setups. With RAG, Qwen-max showed improvements of 15.3% and 14.2% in Tasks 1 and 2, respectively. This indicates that integrating external knowledge enhances the overall accuracy of model responses. In contrast, the effectiveness of CoT varied significantly depending on the task type and model characteristics. For instance, in Task 2, the performance of some models using CoT is even lower than that of the Base configuration. For example, ChatGPT4’s accuracy drops from 39.4% to 36.3%. This decline may be attributed to the increased complexity of Task 2, where relying solely on the model’s internal knowledge is insufficient to meet the reasoning demands.

**Figure 3.**
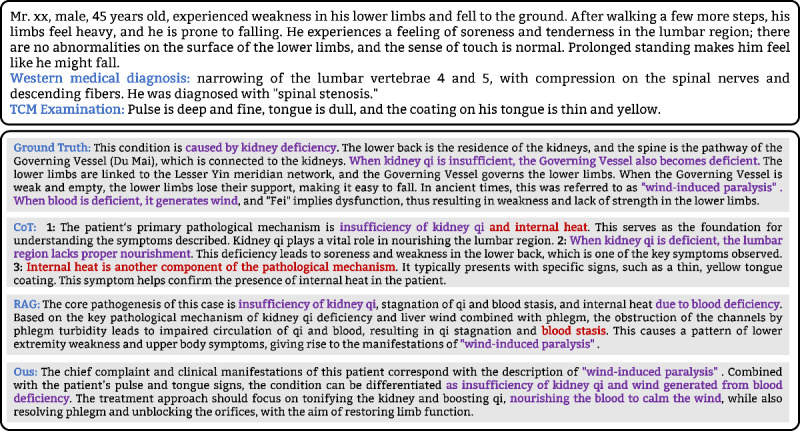
Comparison of results on Task 3: CoT, RAG and our method. CoT: Chain-of-Thought; RAG: Retrieval Augmented Generation.

To explicitly compare the differences between the three methods, we present the results of a case in Task 3, as shown in Figure 3. The ground truth revealed four key conclusions: (1) the condition was attributed to kidney deficiency; (2) kidney deficiency caused weakness in the lower limbs; (3) the condition was identified as wind-induced paralysis; and (4) blood deficiency contributed to the pathology of “wind.”

In the CoT results, the model erroneously inferred the irrelevant factor ’internal heat.’ This discrepancy may stem from the limitations of LLMs’ knowledge, which cannot fully support the accurate processing of complex dialectical reasoning tasks. On the other hand, most of the conclusions from RAG align with the ground truth. But the method incorrectly proposed the diagnosis of ’blood stasis,’ which may be attributed to the increased complexity of the context. Our method successfully derived all key conclusions and effectively mitigated reasoning biases caused by insufficient knowledge, excessive contextual complexity, and challenges faced by other methods.

### Limitations and Future Research

The approach proposed in this paper demonstrates significant advantages in TCM syndrome differentiation thinking. However, there remains room for further refinement when addressing highly intricate and dynamic scenarios in TCM diagnosis and treatment. While the current knowledge base draws extensively from classical literature and clinical case studies, potential biases in coverage may exist. This bias stems from the fact that the literature tends to reflect the medical understanding and insights of the time, which may not be fully compatible with the contemporary clinical context or with different patient populations. In addition, some aspects of diagnostic experience favor specific diagnostic and treatment methods, which can limit their applicability and accuracy in real-world clinical decision-making. Expanding the knowledge base by integrating updated clinical data and interdisciplinary insights could further enhance the LLMs’ ability. Future research should explore dynamic task decomposition strategies to deepen the LLMs’ understanding and improve its adaptability to the nuances of TCM theories.

### Conclusions

In conclusion, this study presents an innovative methodology integrating RAG with instruction fine-tuning to enhance LLMs’ syndrome differentiation thinking ability. Our approach achieved state-of-the-art performance in three core tasks: pathogenesis inference, syndrome inference, and diagnostic suggestion, outperforming both general and TCM LLMs. These findings underscore the importance of incorporating domain-specific knowledge and structured task decomposition to address the unique challenges of TCM reasoning. Although the proposed framework demonstrates significant potential, further advancements are necessary to handle complex, dynamic scenarios and improve conversational abilities.

## Abbreviations

CoT: Chain-of-Thought
LLMs: large language models
RAG: Retrieval Augmented Generation
TCM: traditional Chinese medicine
